# Development of clinical application program for radiotherapy induced cancer risk calculation using Monte Carlo engine in volumetric-modulated arc therapy

**DOI:** 10.1186/s13014-020-01722-0

**Published:** 2021-06-12

**Authors:** Dong-Jin Kang, Young-Joo Shin, Seonghoon Jeong, Jae-Yong Jung, Hakjae Lee, Boram Lee

**Affiliations:** 1 Department of Radiation Oncology, Inje University Sanggye Paik Hospital, 1342, Dongil-ro, Nowon-gu, Seoul, Korea; 2 Proton Therapy Center, National Cancer Center, Goyang, Korea; 3 ARALE Laboratory, Inc, Seoul, Korea; 4 Department of Radiation Oncology, Samsung Medical Center, School of Medicine, Sungkyunkwan University, 81, Irwon-Ro, Gangnam-Gu, Seoul, 06351 Korea

**Keywords:** TPS, Monte Carlo, Secondary cancer risk, Clinical application program

## Abstract

**Background:**

The purpose of this study is to develop a clinical application program that automatically calculates the effect for secondary cancer risk (SCR) of individual patient. The program was designed based on accurate dose calculations using patient computed tomography (CT) data and Monte Carlo engine. Automated patient-specific evaluation program was configured to calculate SCR.

**Methods:**

The application program is designed to re-calculate the beam sequence of treatment plan using the Monte Carlo engine and patient CT data, so it is possible to accurately calculate and evaluate scatter and leakage radiation, difficult to calculate in TPS. The Monte Carlo dose calculation system was performed through stoichiometric calibration using patient CT data. The automatic SCR evaluation program in application program created with a MATLAB was set to analyze the results to calculate SCR. The SCR for organ of patient was calculated based on Biological Effects of Ionizing Radiation (BEIR) VII models. The program is designed to sequentially calculate organ equivalent dose (OED), excess absolute risk (EAR), excess relative risk (ERR), and the lifetime attributable risk (LAR) in consideration of 3D dose distribution analysis. In order to confirm the usefulness of the developed clinical application program, the result values from clinical application program were compared with the manual calculation method used in the previous study.

**Results:**

The OED values calculated in program were calculated to be at most approximately 13.3% higher than results in TPS. The SCR result calculated by the developed clinical application program showed a maximum difference of 1.24% compared to the result of the conventional manual calculation method. And it was confirmed that EAR, ERR and LAR values can be easily calculated by changing the biological parameters.

**Conclusions:**

We have developed a patient-specific SCR evaluation program that can be used conveniently in the clinic. The program consists of a Monte Carlo dose calculation system for accurate calculation of scatter and leakage radiation and a patient-specific automatic SCR evaluation program using 3D dose distribution. The clinical application program that improved the disadvantages of the existing process can be used as an index for evaluating a patient treatment plan.

## Background

Recently, owing to the development of radiotherapy technology such as the multi-leaf collimators (MLC), the use of intensity-modulated radiotherapy (IMRT) and volumetric modulated arc therapy (VMAT) is increasing [1–3]. Especially, VMAT is a treatment technique with the advantage of short treatment time because it provides complex intensity modulated beams by employing dynamic motion of a MLC with rotating gantry. However, VMAT is more likely to induce head-scattered radiation because it uses a higher monitor unit (MU) than conventional therapies such as conformal radiotherapy (CRT) [2, 4, 5]. Therefore, secondary cancer risk (SCR) in VMAT can be increased due to low doses to healthy tissues induced by scatter and leakage radiation from gantry head.

Currently, the SCR receives increasing interest and there are many studies related to the SCR [4, 6–14]. Researches regarding SCR have been reported that SCR increases as the volume of organs receiving radiation increases and as the distance from the source reduces [11, 15]. In Hall et al.’s studies, it was reported that SCR is approximately 1.0–1.8% higher (10 years survival rate) in IMRT than CRT [13, 16]. It is also reported that secondary scattered doses at locations approximately at 20–50 cm from the iso-center were approximately 1.0–5.0 mGy per 1 Gy for planning target volume (PTV) in IMRT [17].

The most commonly used methods to analyze the effects of secondary cancers involve the utilization of human phantoms to measure secondary doses using devices such as the thermo-luminescent dosimeter (TLD) and radio-photoluminescent glass dosimeter (RPLD), and the calculation of doses using commercialized treatment planning system (TPS) [4, 8, 18–20]. Recently, studies have also been conducted to calculate scattered radiation for in-field and out-field, which has a significant impact on SCR using physics-based analysis algorithms [21–24]. Nevertheless, the existing evaluation methods have limitations. First, the method of evaluating by placing the measuring element inside the phantom cannot measure many points, and only the part with holes made for measurement can be measured. Because organ doses are calculated from values of predetermined points due to the structure of the phantom, there is a possibility that it may be evaluated differently depending on the person measuring it [25]. For example, in the case of the thyroid gland, some researchers measure 3 points to evaluate the dose to the thyroid gland, while others measure 5 points to define the thyroid dose. In addition, if the location of the pre-defined hole is not the center of the organ, the dose from the thyroid gland may vary depending on the position selected. Second, the method of measuring the amount of head-scattered radiation according to the distance from the iso-center has little variation in measurement value [26]. However, it is difficult to directly evaluate the patient organ dose because it is a method of measuring the effect of head-scattered radiation [10]. Third, using the TPS dose distribution, the estimated dose that has a large effect on the SCR is not accurate. The reason for the inaccuracy that occurs in TPS is mostly the effect of scatter and leakage radiation in out-of-field [27–32].

The Monte Carlo simulation is another method for SCR analysis, and many studies have been conducted [33–36]. The organ dose estimation utilizing Monte Carlo simulation has the advantage that the dose can be calculated accurately. In a previous study, we have developed a phantom-based dose calculation system for VMAT using GATE Monte Carlo that directly apply scatter dose, difficult to calculate using TPS [37]. And, the system developed in previous studies has been improved to allow direct calculation of patient dose by adding new modules. We verified that the Monte Carlo-based dose calculation system for VMAT calculated the dose distributions for patient-specific treatment plan accurately.

Several studies regarding SCR from stray radiation have reported that it is especially important to predict SCR in order to provide patient’s long-term health information in VMAT treatment with high MU [8, 9]. However, there was no method of patient-specific SCR evaluation program through accurate dose calculation using Monte Carlo in VMAT treatment. In this study, we developed a clinical application program to apply a SCR evaluation process into the workflow of patient treatment. In addition, the automatic quality assurance (QA) system [37] for VMAT based on GATE Monte Carlo simulation which is useful for accurate dose calculation, has added and automated a module that enables calculation using patient specific CT data for direct evaluation in a system developed through previous research. By combining SCR evaluation program and Monte Carlo based automatic QA system for VMAT to improve the accuracy, we intend to apply clinical evaluation of SCR that may be caused by radiotherapy as an indicator of patient treatment.

## Methods

### Patient-specific dose calculation using Monte Carlo engine

We utilized a previously developed clinically available automatic VMAT QA system for this study [37]. The system is designed to re-calculate the beam sequence of equipment and treatment plan using the Monte Carlo engine, so it is possible to accurately calculate and evaluate scatter and leakage radiation, difficult to calculate in TPS [38]. The basic program was designed for QA, so the module was additionally configured to enable dose calculation using patient CT data. The CT contrast phantom (Electron Density phantom 062, CIRS, Norfolk, VA) was utilized to calibrate the electron density and CT number.

Typically, clinical programs perform dose calculation by means of electron density correlations based on the Hounsfield unit (HU) and Compton scattering. In this study, more accurate dose calculations were performed through stoichiometric calibration [39–41]. The mass density and the chemical composition of the tissue were obtained by the method developed by Schneider et al. as a necessary pre-calculation stage for the Monte Carlo-based secondary cancer calculation system [41–43]. The mass density and the chemical composition are shown in Table 1. They are divided into 27 human tissues with CT numbers between − 1050 and 4000 and are interpolated between sections.

**Table 1:** Conversion of Hounsfield Unit (HU) to the chemical composition and weights (%)

HU	H	C	N	O	Na	Mg	P	S	Cl	Ar	K	Ca	Ti	Cu	Zn	Ag	Sn
− 1050	0	0	75.5	23.2	0	0	0	0	0	1.3	0	0	0	0	0	0	0
− 950	10.3	10.5	3.1	74.9	0.2	0	0.2	0.3	0.3	0	0.2	0	0	0	0	0	0
− 120	11.6	68.1	0.2	19.8	0.1	0	0	0.1	0.1	0	0	0	0	0	0	0	0
− 82	11.3	56.7	0.9	30.8	0.1	0	0	0.1	0.1	0	0	0	0	0	0	0	0
− 52	11	45.8	1.5	41.1	0.1	0	0.1	0.2	0.2	0	0	0	0	0	0	0	0
− 22	10.8	35.6	2.2	50.9	0	0	0.1	0.2	0.2	0	0	0	0	0	0	0	0
8	10.6	28.4	2.6	57.8	0	0	0.1	0.2	0.2	0	0.1	0	0	0	0	0	0
19	10.3	13.4	3	72.3	0.2	0	0.2	0.2	0.2	0	0.2	0	0	0	0	0	0
80	9.4	20.7	6.2	62.2	0.6	0	0	0.6	0.3	0	0	0	0	0	0	0	0
120	9.5	45.5	2.5	35.5	0.1	0	2.1	0.1	0.1	0	0.1	4.5	0	0	0	0	0
200	8.9	42.3	2.7	36.3	0.1	0	3	0.1	0.1	0	0.1	6.4	0	0	0	0	0
300	8.2	39.1	2.9	37.2	0.1	0	3.9	0.1	0.1	0	0.1	8.3	0	0	0	0	0
400	7.6	36.1	3	38	0.1	0.1	4.7	0.2	0.1	0	0	10.1	0	0	0	0	0
500	7.1	33.5	3.2	38.7	0.1	0.1	5.4	0.2	0	0	0	11.7	0	0	0	0	0
600	6.6	31	3.3	39.4	0.1	0.1	6.1	0.2	0	0	0	13.2	0	0	0	0	0
700	6.1	28.7	3.5	40	0.1	0.1	6.7	0.2	0	0	0	14.6	0	0	0	0	0
800	5.6	26.5	3.6	40.5	0.1	0.2	7.3	0.3	0	0	0	15.9	0	0	0	0	0
900	5.2	24.6	3.7	41.1	0.1	0.2	7.8	0.3	0	0	0	17	0	0	0	0	0
1000	4.9	22.7	3.8	41.6	0.1	0.2	8.3	0.3	0	0	0	18.1	0	0	0	0	0
1100	4.5	21	3.9	42	0.1	0.2	8.8	0.3	0	0	0	19.2	0	0	0	0	0
1200	4.2	19.4	4	42.5	0.1	0.2	9.2	0.3	0	0	0	20.1	0	0	0	0	0
1300	3.9	17.9	4.1	42.9	0.1	0.2	9.6	0.3	0	0	0	21	0	0	0	0	0
1400	3.6	16.5	4.2	43.2	0.1	0.2	10	0.3	0	0	0	21.9	0	0	0	0	0
1500	3.4	15.5	4.2	43.5	0.1	0.2	10.3	0.3	0	0	0	22.5	0	0	0	0	0
1640	0	0	0	0	0	0	0	0	0	0	0	0	0	4	2	65	29
2300	0	0	0	0	0	0	0	0	0	0	0	0	100	0	0	0	0
3095	0	0	0	0	0	0	0	0	0	0	0	0	100	0	0	0	0

The patient CT data utilized in the Monte Carlo-based SCR evaluation program is converted to analyze 7.5 or metadata format. The patient's CT data was maintained as the 1.27 × 1.27 × 2.5 mm^3^ voxel value of the original raw data for dose calculation. The commercialized TPS performs calculation after reconstructing the pixel values of patient CT data into a preset calculation grid size of 3 mm^3^. In the case of Monte Carlo calculation, after the physical calculation of the dose deposit was completed based on the voxel value of 1.27 × 1.27 × 2.5 mm^3^, it was reconstructed into a pixel size of 2 mm^3^ to represent the dose distribution [44, 45]. Information regarding the number of fields, position of the gantry, field weight, and MLC of the patient treatment plan is converted to a macro file and stored in the system [37]. GATE v8.1, officially released in 2018, was employed for system configuration because it has the advantage of easy geometry configuration and fast calculation time with parallel computing using job split. Optimization was improved compared to the previous study, so the dose calculation time was approximately 10 h per patient [46]. The dose distribution after the dose calculation is stored in a 3D dose distribution DICOM format compatible with TPS.

### Patient-specific calculation of radiation-induced cancer

**Fig. 1:**
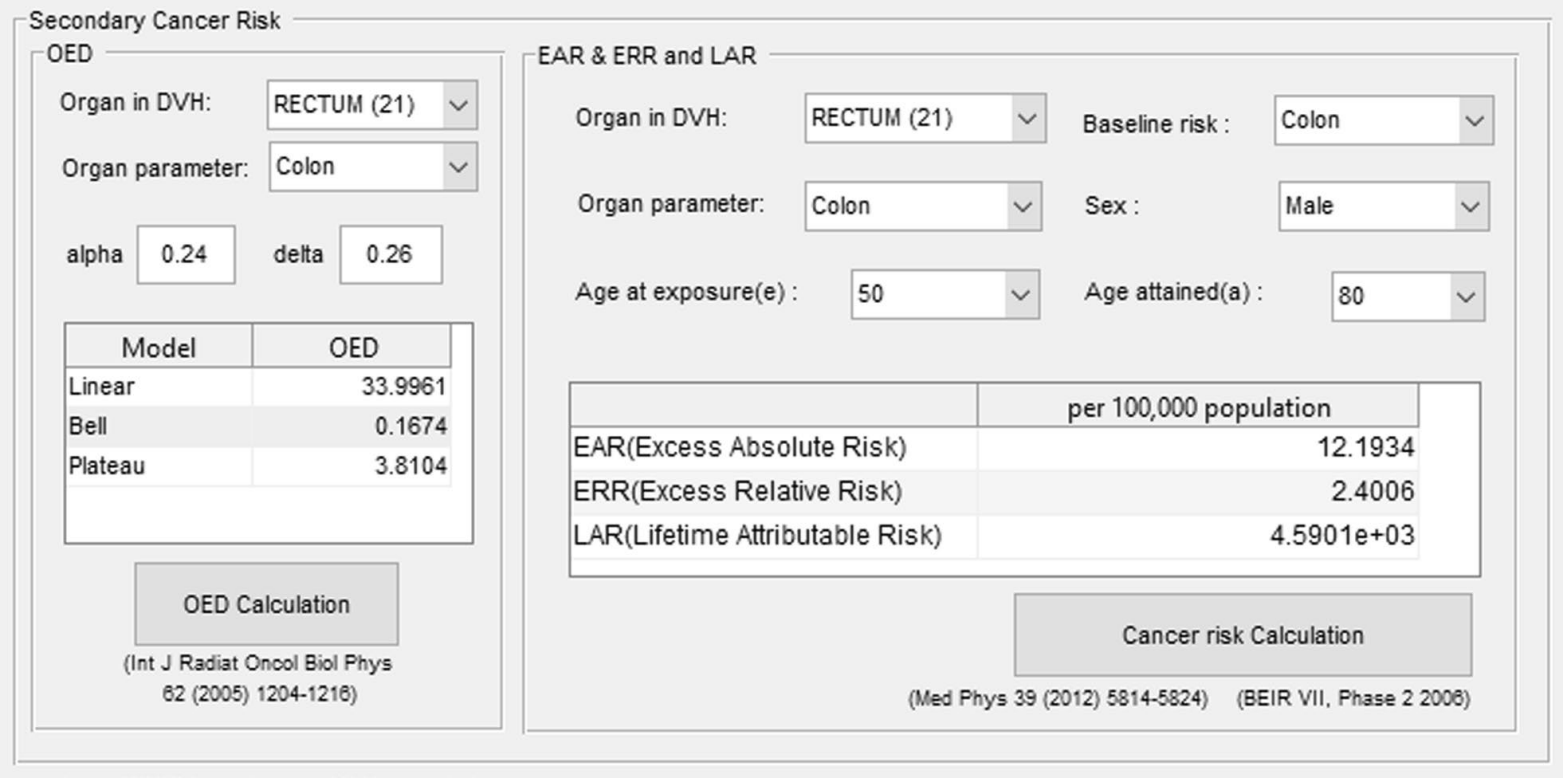
Graphic user interface (GUI) for organ equivalent dose (OED), excess absolute risk (EAR), excess relative risk (ERR), lifetime attributable risk (LAR) calculation of secondary cancer risk (SCR) evaluation program

The automatic SCR evaluation program created with a simple graphical user interface (GUI) using MATLAB (R2016a, MathWorks Inc., Natick, MA, USA) was set to re-calculate the treatment plan with the Monte Carlo engine and analyze the results to calculate SCR. The results calculated by the Monte Carlo engine are first converted into dose-volume histogram (DVH) in the application program. The DVH is a volume-based graph of dose distribution and is often used as the primary data for dose analysis in clinical and research applications [47–49]. The results of organ equivalent dose (OED) are constructed to be calculated based on DVH. The SCR calculation part of program is divided into two categories as shown in Fig. 1: OED and SCR. After selecting the organs and the parameters to be evaluated, the OED was calculated and the results were utilized to sequentially calculate the excess absolute risk (EAR), excess relative risk (ERR), and the lifetime attributable risk (LAR). The organs, gender, baseline cancer risk, age and biological factors for calculating EAR, ERR, and LAR were made up of a selection list to allow easy selection and calculation. The SCR was evaluated using the Biological Effect of Ionizing Radiation (BEIR) VII model [8, 9, 50].

The OED is a dose calculation method to evaluate the effects of SCR caused by radiotherapy. The OED corresponds to a dose response proportional to the population cancer incidence based on the same gender and age. In other words, it is a concept that has the same risk for SCR when having the same OED, and is configured to be able to calculate using 3D dose distribution. The OED has 3 models (Linear, Bell shape, Plateau model) [26]. In the case of SCR, we used a plateau model for specific OED because the linear type of dose–response as in the low dose region does not appear in the high dose region. This is because there is no effect of cancer risk in dead cells due to cell killing in the high dose region.

The OED was calculated as a plateau model by (1) based on tissue dose distribution [13, 51–53]. $V$ is the total volume of the organ for calculating the OED, $V_{i}$ is the corresponding volume, $D_{i}$ is the volume dose, *α* and $\delta$ are dose–response factors of specific organs [54, 55].

1 $$ {\text{OED}} = \frac{1}{V}\sum V_{i} \left( {\frac{{1 - \exp \left( { - \delta D_{i} } \right)}}{\delta }} \right) $$

The EAR is the rate of disease in the exposed population minus the rate of disease in the unexposed population. It means the number of people with secondary cancer caused by exposure to radiation. The ERR is the same as EAR, but is a relative concept. The EAR and ERR of secondary cancer were calculated with (2), the LAR were evaluated based on two calculated EAR and ERR [8].

2 $$ {\text{ERR}}\left( {{\text{D}},{\text{s}},{\text{e}},{\text{a}}} \right){\text{ and EAR}}\left( {{\text{D}},{\text{s}},{\text{e}},{\text{a}}} \right) = {\upbeta }_{{\text{S}}} {\text{Dexp}}\left( {{\gamma e}^{*} } \right)\left( {\frac{{\upalpha }}{60}} \right)^{{\upeta }} $$

The biological parameters utilized in (2) were based on the data obtained from the Hiroshima bombing and the Chernobyl accident [56]. D, s, e, and a denote exposure dose, gender, exposure age; e* = (e−30)/10 for e < 30 and 0 for e > 30, and attained age, respectively [50]. The four parameters are the factors that have the highest effect on the results of the SCR. LAR was calculated from EAR and ERR utilizing (3).

3 $$ {\text{LAR}}\left( {{\text{D}},{\text{s}},{\text{e}},{\text{a}}} \right) = \left( {\mathop \sum \limits_{{{\text{a}} = {\text{e}}}}^{90} {\text{ERR}}\left( {{\text{D}},{\text{s}},{\text{e}},{\text{a}}} \right) \times {\uplambda }_{{\text{I}}}^{{\text{C}}} \times \frac{{{\text{S}}\left( {\text{a}} \right)}}{{{\text{S}}\left( {\text{e}} \right)}}{\text{da}}} \right)^{0.7} \times \left( {\mathop \sum \limits_{{{\text{a}} = {\text{e}}}}^{90} {\text{EAR}}\left( {{\text{D}},{\text{s}},{\text{e}},{\text{a}}} \right) \times \frac{{{\text{S}}\left( {\text{a}} \right)}}{{{\text{S}}\left( {\text{e}} \right)}}{\text{da}}} \right)^{0.3} $$

$\lambda_{I}^{C}$ represents the baseline cancer risk and $S\left( a \right)$ is the survival rate of attained age, and $S\left( e \right)$ is the survival rate of exposure age. $S\left( a \right)/S\left( e \right)$ is the probability of living up to the attained age when surviving the exposure age. The baseline cancer risk was calculated from the annual report of cancer statistics in Korea in 2016 [8, 57]. And for SCR, LAR is a single estimate that can be expressed as one value and is calculated as a weighted average value for ERR and EAR. By the recommendation of the BEIR VII Committee, the weight of 0.7 for the ERR and 0.3 for the EAR were calculated on a logarithmic scale [9].

We set the life expectancy to 90 years and calculated the change in SCR according to the exposure age from 30 to 80 years. The automatic SCR evaluation program based on the GUI is designed to achieve the desired results by changing several parameters using 3D dose distribution. In order to evaluate clinical application of the program, we randomly selected five treatment plans using VMAT that were actually treated. For the evaluation of various treatment sites, five treatment plans were assigned for each disease site of brain, lung, abdomen, prostate and spine.

## Results

**Fig. 2:**
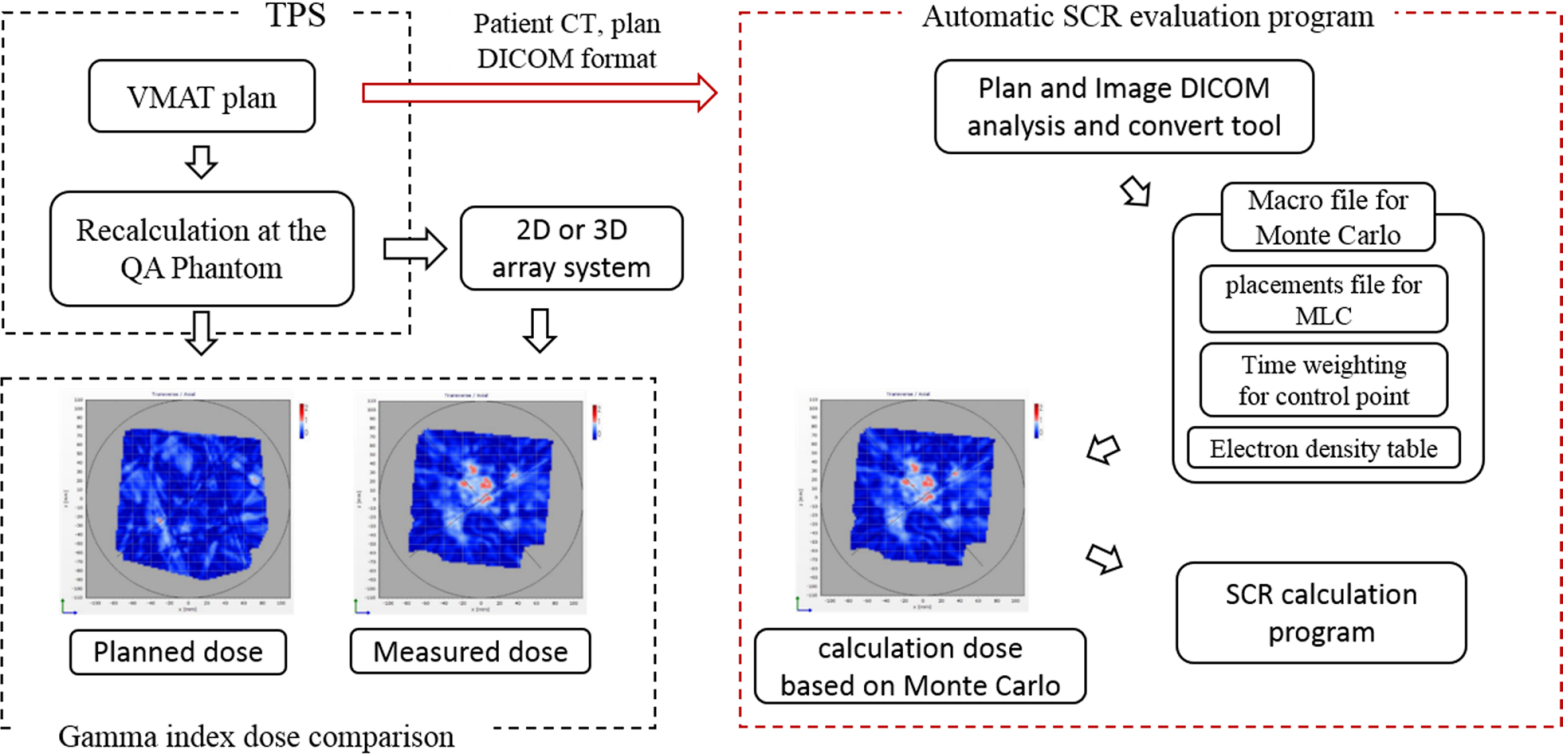
Procedure diagram for the automatic SCR evaluation program based on the Monte Carlo engine. *TPS* treatment planning system, *CT* computed tomography, *SCR* secondary cancer risk, *DICOM *digital imaging and communications in medicine, *MLC* multileaf collimator

The Fig. 2 shows a schematic diagram of the workflow that our developed clinical application program is used in actual clinical practice. The left side is the existing flow and the right side shows the flow when the developed clinical application program is applied. The five treatment plans selected to evaluate the program were calculated step by step according to the Monte Carlo dose calculation system developed in the study. The patient-related data is converted in the program to apply to the re-calculation using the Monte Carlo engine, and the previous results were calculated as the value for SCR by applying each biological parameter. The most time-consuming procedure in the system was the dose re-calculation using the Monte Carlo engine, which took about 10 h. The OED calculation results are shown in Table 2. The OEDs calculated using the Monte Carlo engine were compared with OEDs calculated using the dose from the TPS. The OED results using the Monte Carlo based program developed in this study showed a difference of about 0–13.3% compared to the OED results of TPS. The out-of-field organ located away from the treatment region showed a larger difference in OED calculation.

**Table 2:** Results for OED, EAR and ERR calculation of VMAT plan in MC and TPS

	Organ	OED in MC (Gy)	OED in TPS (Gy)	EAR^a^ (per 100,000 population)		ERR^a^ (per 100,000 population)	
				MC	TPS	MC	TPS
Brain	Rt. Eye	1.5	1.5	9.5	9.4	0.4	0.4
	Brainstem	4.6	4.5	28.8	27.0	1.3	1.2
	Rt. Parotid	0.3	0.3	1.7	1.7	0.1	0.1
	Rt. Lens	1.0	1.0	6.5	6.5	0.3	0.3
Lung	Lt. Lung	3.0	2.8	6.9	6.4	1.0	0.7
	Rt. Lung	3.6	3.5	8.4	8.2	1.2	1.2
	Esophagus	4.4	4.1	27.2	25.3	1.2	1.1
	Heart	1.8	1.8	11.2	11.2	0.5	0.4
Abdomen	Liver	0.7	0.7	0.7	0.7	0.2	0.2
	Cord	4.3	4.4	20.7	21.2	1.9	1.9
	Lt. Kidney	6.3	6.3	39.1	38.0	1.7	1.5
	Rt. Kidney	6.1	6.0	37.7	34.0	1.6	1.4
	Colon	3.4	3.3	10.8	10.0	2.1	1.7
Prostate	Bladder	0.2	0.2	0.2	0.2	0.1	0.1
	Rectum	1.4	1.4	4.4	4.4	0.9	0.9
Spine	Bowel	1.3	1.2	8.0	7.2	0.3	0.3
	Liver	0.2	0.1	0.3	0.1	0.0	0.0
	Lt. Kidney	1.7	1.5	10.7	8.4	0.5	0.4
	Rt. Kidney	1.0	0.9	6.0	3.7	0.3	0.2

*OED* organ equivalent dose, *VMAT* volumetric modulated arc therapy, *TPS* treatment planning system, *Rt* right, *Lt* left, *MC* Monte Carlo, *EAR* excess absolute risk, *ERR* excess relative risk

^a^Caution: the value of EAR and ERR were the maximum values set from exposure age 30 to attained age 90

Table 3 shows the LAR of each organ according to the age difference exposure from the ages of 30–80 years old based on the attained age of 90 years old and varied between 0.017 and 6.294%. The minimum value of 0.017% appeared in the lens of brain disease site and the maximum value of 6.294% appeared in the colon of abdomen disease site. For example, in the case of LAR on the right lens, based on an exposure age 80 to attained age 90, this means that 17 people per 100,000 population is at risk for secondary cancer.

**Table 3:** LAR (%) for organs according to exposure age based on attained age 90 in VMAT plan (per 100,000 population)

	Organ	Age at exposure					
		30	40	50	60	70	80
Brain	Rt. Eye	1.009	0.930	0.829	0.688	0.476	0.188
	Brainstem	0.558	0.490	0.411	0.318	0.201	0.077
	Rt. Parotid	0.186	0.171	0.153	0.127	0.088	0.035
	Rt. Lens	0.125	0.110	0.092	0.071	0.045	0.017
Lung	Lt. Lung	3.411	3.188	2.913	2.453	1.645	0.574
	Rt. Lung	4.152	3.881	3.546	2.986	2.002	0.698
	Esophagus	2.883	2.659	2.369	1.966	1.360	0.537
	Heart	1.185	1.093	0.974	0.808	0.559	0.221
Abdomen	Liver	0.221	0.205	0.183	0.145	0.092	0.033
	Cord	3.754	3.467	3.087	2.581	1.832	0.745
	Lt. Kidney	1.874	1.687	1.410	1.039	0.622	0.192
	Rt. Kidney	1.944	1.751	1.463	1.078	0.646	0.200
	Colon	6.295	5.809	5.118	4.001	2.461	0.861
Prostate	Bladder	0.074	0.066	0.054	0.041	0.030	0.020
	Rectum	2.577	2.406	2.132	1.775	1.391	1.026
Spine	Bowel	0.850	0.767	0.641	0.506	0.381	0.277
	Liver	0.114	0.108	0.097	0.082	0.067	0.052
	Lt. Kidney	0.532	0.507	0.457	0.396	0.332	0.266
	Rt. Kidney	0.299	0.284	0.257	0.223	0.187	0.149

*Rt* right, *Lt* left

**Fig. 3:**
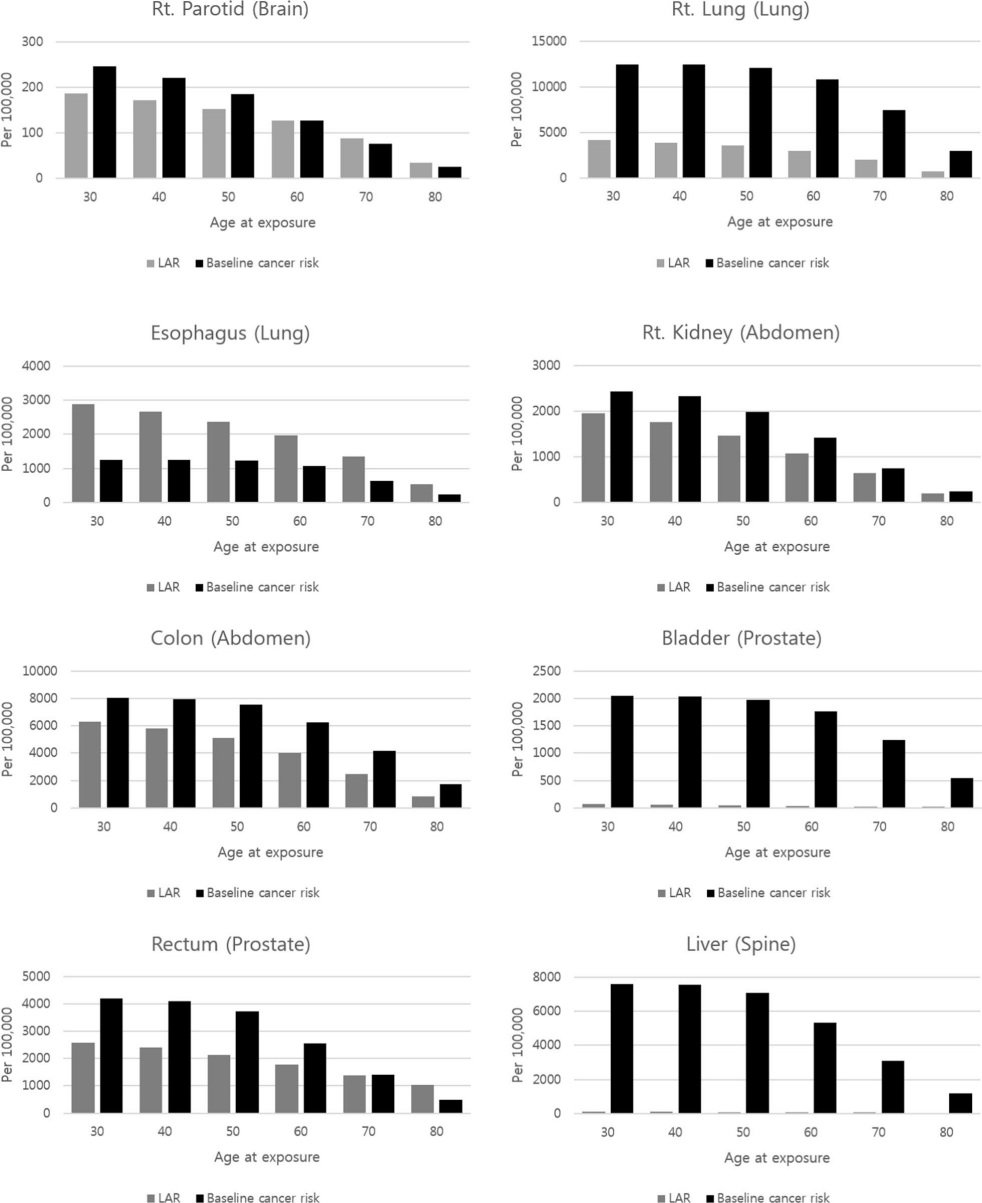
Comparison of LAR and cumulative baseline cancer risk according to exposure age for OAR (treatment site). *LAR* lifetime attributable risk, *OAR* organs at risk

Figure 3 shows the relationship between cumulative baseline cancer risk and LAR with increasing age at exposure. The baseline cancer risk is the rate of cancer incidence by age without the effects of radiation. And it was converted to a cumulative baseline cancer risk to compare with LAR. As with LAR, the cumulative baseline cancer risk was expressed as the sum from exposure age to attained age. By comparing the two values, we tried to easily confirm the effect of secondary cancer caused by radiation. The cumulative baseline cancer risk and LAR expected to be caused by radiation decreased with increasing exposure age. In addition, radiotherapy-induced SCR is relatively high at a young age. The results show that exposure to radiation at a young age is relatively dangerous. Through Fig. 3, we can confirm some things. In radiotherapy of prostate and spine, bladder and liver could be seen that the incidence of secondary cancer caused by radiation was very small than the cumulative baseline cancer risk. This means that compared to other organs, the bladder and liver do not have to consider the effect of secondary cancer. On the other hand, it was found that the SCR of esophagus was significantly higher than cumulative baseline cancer risk in lung radiation therapy. These results suggest that the effects for esophagus should be carefully considered when treating lung site. Of course, these results are only one of the indicators for analyzing the treatment plan, but it does not mean that other organs need not be considered. Depending on the patient, the effects of esophagus may not be significant in lung radiation therapy. So, we think that a SCR evaluation program that can be applied directly to each patient is needed. We should consider the risk of secondary cancer from radiation when evaluating these organs.

The LAR result calculated by the conventional manual calculation method showed a maximum difference of 1.24% compared to the result of the developed clinical application program. We confirmed that the clinical application program proceeded without problems in the procedure for evaluating treatment plans in clinical practice.

## Discussion

The radiotherapy-induced secondary cancer risk has been recognized and evaluated in different studies [4, 6–14]. The developed clinical application program is designed to evaluate SCR caused by radiation through analysis of the patient's treatment plan. However, existing SCR evaluation methods had some the disadvantage in measurement method. The disadvantages include a method of measuring the amount of stray radiation that varies depending on the distance from iso-center, or methods of measuring organ doses by inserting measuring elements such as TLD and RPLD into the human phantom. The method utilizing the point dose measurement device and human phantom has a disadvantage that it is difficult to calculate the accurate organ dose depending on the fixed measurement location [25]. Therefore, dose effect on the volume cannot be accurately evaluated. And the measurement results are standard data using phantoms, actual radiotherapy present high dose difference depending on treatment plan or patient, even with the same location of the disease.

In addition, the method using the TPS has a disadvantage of impossibility to calculate the effects of scatter and leakage radiation, which produces an important effect on SCR. For example, in the case of beam data measurement conducted by commissioning of TPS, 6 MV energy measured a range up to 20.5 cm based on 1.5 cm depth (D_max_) and 10 × 10 cm^2^ field size. Therefore, it is estimated that no dose is present at the outside of 10.25 cm off the central axis in beam data. However, the actual scatter and leakage radiation exist, and the results of the Monte Carlo simulation indicate that a dose of approximately 1% is calculated even at a location 13.0 cm from the central axis. And at the same position, TPS showed a value of 0.4%. When actually measured, a value over 1% was measured at the same location. For these reasons, in the case of radiotherapy in the same disease site, the OED value of the organ may vary depending on the patient or the treatment plan. In addition, OED showed a greater difference between Monte Carlo and TPS as the distance from the treatment field increased. As the distance from the treatment region increases, the difference in OED can be considered for the following reasons. First, it is the limitation of the application for TPS commissioning as described above. And second is the use of OED based on the plateau model. The plateau model is a biological model that explains that the dose and risk are not proportional to the dose above the threshold, as in low dose. Increasing the distance from the treatment region means a difference in the low dose, and thus has a greater effect.

We believe that a patient-specific SCR evaluation program that can be applied directly to a patient is required because the effect of the dose is different for each patient to predict results with one or two measurements. The difficulty in system configuration was plan re-calculation utilizing the Monte Carlo engine, and required approximately 10 h to evaluate the patient to optimize the effects from scatter and leakage radiation. However, the most time-consuming Monte Carlo dose calculation is constantly being improved. Currently, general clinical application is difficult in workflow due to time limit, so it can function as a prototype program that is applied to special treatment such as stereotactic radio surgery (SRS), stereotactic body radiation therapy (SBRT) or pediatric treatment. We are now using the 88 node high performance computing (HPC) system we designed, but we are planning to upgrade the HPC system so that it can be calculated within 3 h soon. After that, the program is expected to be clinically applicable to the VMAT plan.

In SCR evaluation, it is important to perform a biological evaluation of the effect of the overall OAR volume. There have been many studies on neutrons and scatter radiation from gantry head to evaluate the effects of secondary cancer [58, 59]. The calculation method of secondary cancer by scatter radiation from gantry head without evaluating the volume of a specific organ may have high uncertainty. The human phantom and Monte Carlo calculation are required to evaluate the volume of a specific organ. So, we developed a clinical application program that can evaluate organ doses for individual patients. We have constructed and evaluated a clinical patient-specific automatic SCR evaluation program that can accurately calculate scatter and leakage radiation. The dose calculation of the treatment plan for the program test showed that the scattered dose difference between the Monte Carlo calculation system and the TPS was larger as distance increased from the treatment region. To verify the accuracy of the calculated SCR evaluation, no significant difference within 1.24% compared with the manual calculation method was observed when compared with the results using the excel sheet from the previous study [4, 9]. The disadvantage of the existing method is that the procedure is complex and must be checked manually to verify the accuracy of the calculation results. In addition, it is necessary to observe the effect of changes in the region to be evaluated and various factors, but there are many parameters to be checked in the sheet because the related equation is complex. And it takes a lot of time because it has to go through a process such as going through another person's verification to confirm the calculated data. For this reason, the existing system had many difficulties in workflow to be applied to each patient in clinical trials.

The automatic patient-specific SCR evaluation program has the advantage of enabling accurate evaluation of scatter and leakage radiation from gantry head for each patient treatment plan and convenient SCR evaluation for various parameter changes.

## Conclusions

The effects of radiotherapy-induced secondary cancer risk have been evaluated in several studies [4, 6–14], and the development of evaluation tools for treatment plan to help with various clinical decisions has been carried out. To improve the disadvantage of the previous study, the system combines Monte Carlo dose calculation system and SCR evaluation program. The automatic SCR evaluation program can be useful for OED calculation and SCR evaluation for each patient.

By utilizing the developed clinical application program, we plan to conduct a patient-specific SCR evaluation and further study how to establish the means for a SCR database for patients in South Korea. The data will be used as a new evaluation index for SCR in South Korea.

## Data Availability

Please contact author for data requests.

## Abbreviations

MLC: Multi-leaf collimators
IMRT: Intensity-modulated radiotherapy
VMAT: Volumetric modulated arc therapy
CRT: Conformal radiotherapy
MU: Monitor unit
SCR: Secondary cancer risk
PTV: Planning target volume
TLD: Thermo-luminescent dosimeter
RPLD: Radio-photoluminescent glass dosimeter
TPS: Treatment planning system
CT: Computed tomography
QA: Quality assurance
HU: Hounsfield unit
GUI: Graphical user interface
BEIR: Biological effect of ionizing radiation
OED: Organ equivalent dose
EAR: Excess absolute risk
ERR: Excess relative risk
LAR: Life-time attributable risk
HPC: High performance computing
